# From entrepreneurial cognition to entrepreneurial intention and behavior: The case of higher educational institutions in China

**DOI:** 10.3389/fpsyg.2022.1045050

**Published:** 2022-11-09

**Authors:** Su Chen, Wenbin Shen, Xinyu Tan, Rongzhi Liu

**Affiliations:** ^1^School of Accounting, Wuhan Textile University, Wuhan, China; ^2^School of Management, Zhongnan University of Economics and Law, Wuhan, China

**Keywords:** entrepreneurship, higher educational institutions, cognition, intention, behavior

## Abstract

Entrepreneurship has been called “high quality employment” in China. Therefore, universities have paid more attention to entrepreneurship education, which is a crucial element for entrepreneurial success. Based on the theory of “informed intentions” planned behavior and dual cognitive processing theory, this manuscript studies the relationship among entrepreneurial cognition, entrepreneurial intention, recognition perception of university entrepreneurship education, and entrepreneurial behavior from the perspective of mass innovation and mass entrepreneurship in China. The hypotheses are tested using a hierarchical linear regression model based on data from 786 valid questionnaires from more than 400 universities across China. This study finds that student’s entrepreneurial cognition positively affects their entrepreneurial behavior, and entrepreneurial intention plays a mediating role by positively strengthening the relationship between these two, and that the recognition perception of university entrepreneurship education strengthens the positive relationship between entrepreneurial intention and entrepreneurial behavior. These findings provide a new perspective and framework for studying the entrepreneurial cognitive education of university students, and they have certain practical implications for the reform of entrepreneurship education in China.

## Introduction

In recent 20 years, with the rapid development of Internet economy across the worldwide, more and more people from all walks of life have joined the entrepreneurial army. According to relevant statistics, China’s entrepreneurial activity is now foremost in the world, but the quality of entrepreneurship is not high, the success rate is relatively low, there are fewer highly educated entrepreneurs, and they are still concentrated in low-tech industries (China Entrepreneurship Research Center, 2013). A large-sample survey focused on entrepreneurship showed that 64.9% of Chinese college students have entrepreneurial ideas, but the proportion of successfully achieving the business goal is less than 20%. Also, their income is relatively low, nearly 70% of them have an average monthly income of less than 3,000 yuan, and 17.3% of the entrepreneurial projects run at a loss (Maxis Institute, 2014). Therefore, it is impossible to achieve entrepreneurial success with just entrepreneurial enthusiasm but no entrepreneurial ability (i.e., accurate understanding of entrepreneurial theory, direction, objectives, risks, implementation process, and sustainability evaluation). For successful entrepreneurship and social value growth, it is crucial to cultivate and improve individual entrepreneurial ability (Audretsch, 2007), which is why major countries have prioritized the cultivation and development entrepreneurial capabilities (Chen et al., 2014).

At present, under the guidance of a policy of the Chinese government- “Basic Requirements for Entrepreneurship Education and Teaching in Ordinary Undergraduate Universities,” most colleges and universities in China have consciously established theoretical and practical courses such as “Entrepreneurship Management” and “Entrepreneurship and Employment Guidance” to guide the entrepreneurial cognition and practice for college students. Taking the city of Wuhan in the province of Hubei as an example, 96% of colleges and universities there have established a course on entrepreneurship management, and government departments at all levels have implemented a series of preferential policies of Wuhan City Government- “Millions of College Students to Stay in Wuhan for Starting a Business” under the guidance of “Mass Entrepreneurship and Innovation.” However, according to data from the Entrepreneurship Guidance Center of the Human Resources and Social Security Bureau, nearly 12% of Chinese students have the intention to start a business, while only 2% actually practice, which is in sharp contrast to the stable proportion of 20–30% in Western developed countries (Rongzhi and Hu, 2011). In recent years, many young students have been involved in entrepreneurship because of (i) the increasingly severe employment situation, (ii) the examples and influences of successful entrepreneurs, and (iii) the implementation of a series of preferential policies for innovation and entrepreneurship by university students. Therefore, the following questions arise. What are the common characteristics of university students will choose to start a business? What factors will affect and promote college students’ entrepreneurial intention (EI)? How do university students perceive our entrepreneurship education? How to accurately understand the role of EI in the process of individual entrepreneurial behavior (EB)? How entrepreneurship education in universities should be reformed and innovatively to promote the benign promotion and sustainable development of students’ entrepreneurial intentions and behaviors, so as to help students’ innovation and entrepreneurship could further successfully going out of universities, stepping into society, participating in the business, and realizing entrepreneurship in a real sense has been still a hot issue concerned by the academic circle and relevant departments.

## Research objectives

### Entrepreneurial cognition and entrepreneurial behavior

At the beginning of the 21st century, scholars beyond China pointed out the influence of entrepreneurial cognition (EC) on EB. Mitchell et al. (2002a) defined EC as “the knowledge structure used by entrepreneurs for evaluation, judgment and decision-making in the process of opportunity evaluation and entrepreneurial growth,” and while studying entrepreneurship, Li et al. (2013) found that EC has a significant impact on entrepreneurial decision-making. Therefore, many colleges and universities in China now have an entrepreneurship management course for EE: this provides college-student entrepreneurs with theoretical knowledge about and practical experience of entrepreneurship through diversified education modes such as innovation and entrepreneurship competitions, social practice, enterprise practice, and project simulation, which are very important for improving their EC (Li et al., 2013). Therefore, the first purpose of the present study is to explore the specific impact of EC on EB.

### Entrepreneurial cognition and entrepreneurial intention

Most empirical studies in China and elsewhere have shown that EI plays an important and prerequisite role in the entire process of individual EB (Xiu’e and Kun, 2016), and as a classic concept in psychology, that of “informed intentions” is often used in the moral education of college students. Entrepreneurship is a typical planned behavior. As an exogenous factor, entrepreneurial education (EE) in colleges and universities has been concerned with whether it is possible to enrich the self-awareness of entrepreneurship among college students by teaching entrepreneurial knowledge, thereby stimulating EI and encouraging EB (Bird, 1988). Also, researches have noted that entrepreneurship—like other disciplines—can be recognized and understood through learning (Bird, 1988). Many previous studies have also shown that EE has a certain positive impact on improving people’s EI or entrepreneurial ability. Based on empirical research, the positive influence of entrepreneurship education on college students’ entrepreneurial attitude and entrepreneurial self-efficacy has been discussed (Peter and Kennedy, 2003; Fayolle et al., 2006; Souitaris et al., 2007). Also, EE has a positive and significant effect on the formation of students’ EI and entrepreneurial motivation has been reasoned (Li and Huiming, 2010). And via a questionnaire survey, it pointed out that EE has not only a direct effect on EI but also a significant impact on it with a generalized entrepreneurial attitude as an intermediate mechanism (Li and Huiming, 2010; Xiang and Lei, 2014). From the discussion above, it could be concluded that EE in colleges and universities actually involves cognitive education about entrepreneurship-related knowledge to stimulate the EI of college students through systematic cognitive education and then stimulate their EB. Therefore, another purpose of the present study is to explore whether EC also follows the psychological law of “cognition–emotion–intention–behavior,” i.e., whether EC and EI are correlated positively.

### Recognition of college entrepreneurship education and entrepreneurial intention and behavior

EI plays an important role in the whole process of the individual entrepreneurship of college students. However, from the perspective of the pre-factors affecting EI, it is not only determined by the EC acquired from EE in colleges and universities but also may be influenced by individual subjective attitudes, subjective norms, and perceived behavioral control (Heuer and Kolvereid, 2014). In recent years, some studies have found that (i) EE has little effect on the entrepreneurial ability of college students and (ii) EE has no or even negative impact on EI (Oosterbeek et al., 2010; Rongzhi and Hu, 2011). (iii) no significant difference in the impact of whether students had received EE in the three aspects of innovation ability, risk-taking ability, and desire for success (Li and Huiming, 2010). Further, some also explored the moderating role of entrepreneurial competencies between use of e-commerce and SME performance (Hussain et al., 2022). In general, there has been much academic research over the years into the relationship among EI, EC, entrepreneurial ability, EE, and EB, but claims about specific positive or negative relationships have been controversial. As a new part of higher education, the present study reasons that the mode adopted by EE and the corresponding education quality satisfaction can have a positive or negative impact on college students’ EC, and colleges and universities can continuously improve the learning mode of EE and enhance recognition of college EE, so as to improve the EC level of college students with EI and help them to effectively carry out EB and further to achieve entrepreneurial success, which means that EI is the internal factor of college students’ EB, EE is the external factor of their EB, and the EC obtained from EE is an important driving factor to stimulate EI and practice EB. Therefore, the third purpose of this manuscript is to introduce recognition of college EE as a regulating variable to explore whether it plays a role in the impact of college students’ EC on their EI and EB under the current national entrepreneurial upsurge.

## Research design

### Hypotheses development

#### Entrepreneurial cognition and entrepreneurial behavior

The *Psychology Volume of Cihai* (a well-known Chinese lexicon and character dictionary) defines “cognition” as the psychological process of individual cognition and understanding of things, i.e., the activities of human beings to recognize objective things and acquire knowledge: it is embodied in the process of processing and applying the acquired information, mainly including sensing, perception, attention, imagery, learning, memory, verbal problem solving, and decision-making, etc. The term “cognition” is used in many fields: in educational psychology, researchers often use it to express the information-processing view of individual mental activities of students, including processes such as perception, memory, learning, speech, thinking, and problem-solving; in behavioral psychology, researchers often use it to explain people’s attitudes, behaviors, attributions, and group dynamics, etc. Meanwhile, in the *Psychology Volume of Cihai*, “behavior” refers to the sum total of all reflections of the organism to the situation, including all internal and external, physiological and psychological reflections. Different branches of psychology study behavior from different angles: physiological psychology studies the physiological mechanism of organism behavior mainly from the perspective of hormones and nerves, while cognitive psychology studies it mainly from the perspective of information processing (Ning, 2017). In summary, combined with ternary interactive determinism, there is arguably a natural connection between cognition and behavior, i.e., some specific behavior of an individual is determined by the interaction of three factors, such as the external environment, individual cognition, and individual behavior. This ternary interaction makes the three factors not only act directly on individual behavior but also interact with each other and then further influence individual behavior.

In the theoretical and practical perspectives of pedagogy, the importance of cognition has never been overlooked. For example, in the ancient Chinese educational classic *Xue Ji*, the so-called “separation from the scriptures to distinguish one’s aspirations” and “knowing the rest of a kind by analogy” contains the original spirit of “cognition.” For Western intellectuals, the pursuit of rational spirit nurtured by the ancient Greek philosophers laid the foundation for the overall development of Western philosophy, culture, and education and thus naturally laid out the prototype of cognitive learning and teaching. And, the Learning and Teaching for Understanding (LTFU) project launched by the Harvard Graduate School of Education in the 1990s promoted cognitive-oriented learning from teaching theory to the forefront of the world’s educational research. Furthermore, according to dual cognitive processing theory, individual behavior is determined by the interaction between automated processing and controlled processing which will influence personal cognition, intention and behavior (Miles, 2021).

EC originates from the development of traditional social cognition concepts by entrepreneurial scholars, which refers to the knowledge structure that individuals use to estimate, evaluate, and make decisions in the evaluation of opportunities and the creation and growth of enterprises (Mitchell et al., 2002a,b). Therefore, EC is a knowledge structure and thinking process, as well as a reflection of entrepreneurial ability, and the cognitive processing includes single and dual cognitive perspective. Many researchers have been pay more attention single procession of EC. Scholars both in and beyond China are yet to achieve consensus on the definition of EB, which is divided mainly into two different views: first, from a broad perspective, it is considered that the whole process of the establishment, survival, growth, and development of new enterprises belongs to EB; second, from a narrow perspective, EB refers only to the process from entrepreneurs’ perception of entrepreneurial opportunities to the integration of entrepreneurial resources to the final establishment of the enterprise (Liu, 2018). In summary, based on the main objects of the present study, it can conclude that college students’ EB refers to the dynamic behavior process in which they as individuals or groups make continuous efforts in team building, opportunity search, market evaluation, fund preparation, business decision-making, and other links to create new enterprises to provide innovative products or services based on their EC. This manuscript reason that EC can positively affect college students’ EB in the following ways.

First, EC is an important part of college students’ EE. It is the knowledge structure used by college-student entrepreneurs to evaluate, judge, and make decisions in the process of opportunity evaluation and the growth of new start-ups (Mitchell et al., 2002a,b). Compared with individuals who lack entrepreneurial awareness, individuals with entrepreneurial awareness can give full play to their advantages after having relevant knowledge, and they will control the entrepreneurial situation from a long-term perspective, constantly surmounting obstacles and solving difficulties until they reach the goal, thus providing a motivation basis for actively implementing EB and actively dealing with uncertain factors in the entrepreneurial process (Ning, 2017).

Second, entrepreneurship is an innovative pioneering behavior, and it is a complex and challenging task to turn innovative ideas into entrepreneurial achievements. It requires entrepreneurs to have systematic, comprehensive, and in-depth knowledge reserves. Based on social cognition theory, Bandura (1986) reasoned that EC directly affects the occurrence of individual thinking, motivation, and EB. Higher EC means more entrepreneurial knowledge and information reserves. When individuals have more and more reserves and channels to obtain knowledge and information, they can deepen their understanding of entrepreneurship itself and self-cognition; meanwhile, active EC can win more learning and practice opportunities for entrepreneurs, enrich the diversified knowledge of EC, make more entrepreneurial choices and decisions, and lay a knowledge and ability reserve for improving the success rate of entrepreneurship, so as to stimulate individual EB (Bird, 1988). Therefore, we propose the following hypothesis.

*H1: EC is positively associated with EB.*

### Intermediary role of entrepreneurial intention

According to the theory of planned behavior, the level of entrepreneurial consciousness and attitude of entrepreneurs determine their preparation before starting a business and whether they have EI. EC is the knowledge structure refers to the entrepreneur to fully understand and affirm the entrepreneurial idea and then put it into action. A high level of EI directs the attention and behavior of entrepreneurs to a specific goal and reinforces the idea. On the contrary, a low level of EC will cause potential entrepreneurs to feel confusion, anxiety, self-denial, and even fear in the face of the many uncertainties and unknown risks that can occur in entrepreneurship, and they may deny and give up entrepreneurship from an emotional perspective, thereby preventing or even terminating the formation of EI. Entrepreneurs who pursue a high level of EC often show individual characteristics of high sense of responsibility and strong affinity, as well as positive and extroverted and hard-working emotional tendencies. These characteristics and tendencies can better stimulate EI and enthusiasm (Chengxu, 2019). Therefore, we propose the following hypothesis.

*H2: EC is positively associated with EI.*

According to the planned-behavior theory of “cognition–emotion–intention–behavior,” from the perspective of psychology, EI is actually a subjective emotional attitude adopted by entrepreneurs in entrepreneurial activities. The emotional attitude of an entrepreneur is the premise for them to perform entrepreneurial activities. When an entrepreneur believes that the entrepreneurial opportunity and environment are mature, they will put the EB into action based on psychological factors such as cognition and emotion (Shi, 2016). For entrepreneurs, EI is the inner driving force for EB. EI can give strong spiritual support to new ventures and help entrepreneurs put their self-confidence into entrepreneurial activities and wish to realize its own value through actual entrepreneurial activities. When an entrepreneur chooses to start a business, their own conditions, resources, cognition of entrepreneurship, and risk identification ability all have an impact on the entrepreneur. When an entrepreneur starts their entrepreneurial activities, their own EI can provide them with rational entrepreneurial goals, so that they can choose and rationally use and expand their own resources in the entire process of entrepreneurial activities. The continuous changes in self-cognition and entrepreneurial environment will influence or guide EI, which will further be helpful for the achievement of entrepreneurial goals (Chengxu, 2019). Therefore, we propose the following hypothesis.

*H3: EI is positively associated with EB.*

Achievement goal theory provides a new theoretical perspective for exploring the relationship among EC, EB, and EI. On one hand, in the face of highly complex and uncertain entrepreneurial challenges, entrepreneurs with high-level EC can conduct serial processing via their existing knowledge and cognition level and combine certain logical reasoning to make a quick judgment on entrepreneurial opportunities and clarify the effective generation of EI, so as to form favorable entrepreneurial resources and conditions. Meanwhile, they can grasp effective information from limited or mostly useless information in the process of analyzing entrepreneurial opportunities, resources and challenges with their acquired entrepreneurial cognition. Therefore, they could get a much clearer new understanding of the entrepreneurial process, and then innovated points would be refined on the basis of “re-cognition” which would again constantly improve their own EI. And then the enhanced EI would also tends to promote EB (Wu and Ma, 2020). On the other hand, after learning the effective integration and rational use of entrepreneurial resources, an entrepreneur is more likely to gain a sense of achievement and satisfaction, thereby further increasing their EI. This increased EI also promotes their advanced EB again (Fei-Fei, 2018).

Combining the discussions of H1 and H2, we suggest that EI plays a mediating role between EC and EB, and we propose the following hypothesis.

*H4: EI mediates the relationship between EC and EB.*

### Mediating effect of recognition of university entrepreneurship education

The earliest appearance of EE can be traced back to the first course offered by Professor Myles Mace of Harvard Business School in 1947, and EE developed rapidly thereafter. And the concept of EE was first formally proposed by UNESCO at the International Symposium on Education for the 21st Century held in Beijing in November 1989: “Entrepreneurship education, broadly defined as the development of pioneering individuals, is equally important to those who earn a salary, because employers or individuals are placing increasing emphasis on employer initiative, risk-taking, entrepreneurial and independent work ability, and technical, social, and managerial skills.” In Ning (2017) further elaborated the complete concept of EE, i.e., it includes two aspects: job search and creation of new jobs. Since then, educational circles and experts both in and beyond China have paid great attention to and thought about EE, and it has also begun to be exploratively popularized and developed in colleges and universities both in and beyond China (Ning, 2017).

Based on previous research, the core contents of EE can be defined as follows: (i) individual college student or teams of college students as the may object of education; (ii) colleges and universities as the main entity, government, society, industry, enterprise, family as the complemented entities (iii) including basic education, higher education and continuing education system (iii) deeply develop the educates has a series of knowledge, ability and character related to their employment and entrepreneurship; (iv) educating purpose is to help educates to plan their career freely, and further to help them to start their own business when certain preparation has been already done, which will not only provide new opportunities for themselves and others, but also make their own contributions to national economic growth and development (Ning, 2017). EE among college students has strong practical significance and theoretical guiding significance. It cannot only reposition the educational function but also deepen the nature and laws of education. EE is an effective way to train educated people to innovate in social economy, culture, and other fields and to meet the real needs of society and open up new development space. In recent years, driving employment through entrepreneurship has been the direct goal of promoting EE in China.

Many studies based on planned-behavior theory have shown that EE and EI are closely related, i.e., EE can promote the improvement of individual EI. On one hand, EE can improve the entrepreneurial awareness of individual college students by imparting basic entrepreneurial knowledge, thereby deepening EC and stimulating EI; on the other hand, through practical education (e.g., writing entrepreneurship plan, participating in entrepreneurship competitions, joining in social practice, etc.), EE can improve the entrepreneurial practical skills and personal sense of achievement of college students or groups and further promote their re-cognition of starting a business process, and then once again clarify and strengthen their own EI (Xiu’e and Kun, 2016). Based on the teach ability theory of EE, scholars both in and beyond China have affirmed the teach ability of entrepreneurship and pointed out that EE can improve individual EB. According to (Tan et al., 2015), the quality of and satisfaction with EE in colleges and universities will impact college students’ EC, so as to encourage or to block them to effectively carry out EB and achieve entrepreneurial success. Therefore, we reason that recognition of college EE will moderate the relationship between EC and EI, and therefore we propose the following hypotheses.

*H5a: The recognition perception of college EE will strengthen the positive relationship between EC and EI.*

*H5b: The recognition perception of college EE will strengthen the positive relationship between EC and EB.*

**FIGURE 1:**
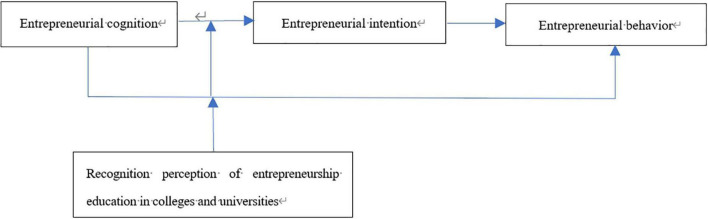
Theoretical framework of present study.

The theoretical model of the present study is shown schematically in Figure 1.

### Study design

#### Sample

In this study, a questionnaire was completed by university undergraduate students. In total, 1200 copies of the questionnaire were sent to nearly 400 colleges and universities across China, and 1,088 were returned, giving a return rate of 90.67%. Of the returned questionnaires, 302 with inconsistent and/or incomplete responses were discarded, and the remaining 786 valid questionnaires were retained.

Of the respondents for those 786 questionnaires, men accounted for 36.01% and women 63.99%, with an average age of 20. The proportions of freshmen, sophomores, juniors, and seniors were 30.66, 35.62, 21.25, and 12.47%, respectively. Regarding the regions containing the colleges and universities, the proportions were as follows: South China (14.76%), Southwest China (8.78%), North China (9.54%), Northeast China (3.44%), East China (11.32%), central China (48.47%), and Northwest China (29%). From most-studied to least-studied, the respondents majored in management, economics, engineering, science, others, literature, education, art, law, philosophy, and history. At the time of responding, non-entrepreneurs accounted for 95.93% and entrepreneurs accounted for 4.07%. The proportion of respondents with family members who had (resp. did not have) entrepreneurial experience was 32.06% (resp. 67.94%). The proportion of those without entrepreneurial experience was 67.94%,those who had not participated in entrepreneurship competition account for 67.18, 25.7% had participated in a start-up competition, and the proportion of those who had participated in entrepreneurship competition several times is 7.12%.

#### Variables measurement

The scales used in this study were all mature ones from the literature and were measured on a five-point Likert scale (5 = strongly agree, 4 = agree, 3 = not sure, 2 = disagree, 1 = strongly disagree).

The scale developed by Mitchell et al. (2002a,b) was used to measure EC. It involved 10 topics, such as “I have entrepreneurial-related interpersonal and wealth networks” and “I have special products or services,” and the value of Cronbach’s α for the scale was 0.873.

A scale adapted from that developed by Yu and Zeng (2010) and Ning (2017) was used to measure EB. It involved five topics, including “I am willing to spend time and energy to prepare for entrepreneurship,” “I have built the interpersonal network required for entrepreneurship,” and other topics reflect directly the entrepreneurial effect, and the value of Cronbach’s α for the scale was 0.877.

The scale developed by Linan and Chen (2009) was used to measure EI. It involved five topics, such as “My career goal is to become an entrepreneur” and “I am determined to establish a company in the future,” and the value of Cronbach’s α for the scale was 0.939.

The scale developed by Jingwei (2013) was used to measure the recognition perception of EE in colleges and universities. In involved nine topics divided into personal factors and school factors, such as “I take the initiative to take courses on EE and attend entrepreneurship lectures” and “The degree to which university leaders attach importance to EE,” and the value of Cronbach’s α for the scale was 0.837.

Moreover, gender, grade, study major, and school area are listed as control variables.

## Data analysis and results

### Homologous variance test

In this study, Harman’s single-factor test method was used to test statistically whether there was any common-method variance, and exploratory factor analysis was conducted on all variables involved in the questionnaire. The first principal component obtained without rotation accounted for 38.341% of the load, and this being less than 40% indicated that a single factor could not explain most of the variation. Therefore, the impact of common-method variance in this study is within an acceptable range.

### Confirmatory factor analysis

The software AMOS 21.0 was used to conduct confirmatory factor analysis to test the discriminant validity of EC, EI, EB, and recognition of college EE as variables, and the results are given in Table 1. Comparing the model fitting indexes of the four models shows that the four-factor model fits the best: the root mean square error of approximation (RMSEA) is 0.11, χ^2^/df is 10.99, the Tucker–Lewis index (TLI) is 0.88, and the comparative fit index (CFI) is 0.91. The results of the confirmatory factor analysis show that the discriminant validity of the variables in this study was good.

**TABLE 1:** Results of confirmatory factor analysis for discriminant validity.

Model	TLI	CFI	RMSEA	*χ^2^*	df	*p*	χ^2^/df	Model comparison test *χ^2^* difference df difference	
Four-factor model 0 (benchmark model)	0.88	0.91	0.11	747.07	68	0	10.99		
Three-factor model 1-xm	0.85	0.88	0.13	972.98	71	0	13.70	225.90**	3
Three-factor model 2-my	0.81	0.85	0.14	1207.96	71	0	17.01	460.88***	3
Two-factor model 1-xy-mw	0.84	0.87	0.13	1103.35	73	0	15.20	356.28**	5
Two-factor model 2-xw-my	0.81	0.85	0.14	1238.75	73	0	17.00	491.68**	5
Single-factor model 6	0.7924	0.8312	0.1503	1386.5882	74	0	18.7377	639.5137**	6

x, m, y, w refer to the independent variable, mediator variable, dependent variable, and moderator variable, respectively; xw refers to the synthesis of the independent variable and moderator variable into one factor; xw, my refer to combining x and w, m and y into two factors. **p < 0.001 (two-tailed test).

The *p*-values in the above table are all significant, indicating that the validity of the questionnaire is in a significant and acceptable range. Four-factor model: EC; EI; EB; recognition and perception of EE in colleges and universities. Three factor model: EC + EI; EB; recognition perception of EE in colleges and universities. Two-factor model: EC + EI + EB; recognition perception of EE in colleges and universities. Single factor model: EC + EI + EB + recognition perception of EE in colleges and universities.

### Reliability test

SPSS 24.0 was used to analyze the collected data statistically, and the analysis values are given in Table 2. The measurement reliability is evaluated by the reliability coefficient, and the two are in direct proportion. A reliability coefficient between 0.70 and 0.80 is acceptable, and a scale or questionnaire with a reliability coefficient greater than 0.80 is relatively good. In Table 2, the reliability coefficient of each of the four variables is greater than 0.8, meaning that the reliability is good. Therefore, correlation analysis can be carried out as the next step.

**TABLE 2:** Reliability results.

Variable	Question nos.	Cronbach’s α	No. of questions
Entrepreneurial cognition	1–10	0.873	10
Entrepreneurial intention	11–15	0.939	5
Recognition perception of entrepreneurship education in colleges and universities	21–31	0.837	11
Entrepreneurial behavior	16–20	0.877	5

### Correlation analysis of variables

SPSS 23.0 was used to analyze the correlation between variables, and the analysis results are given in Tables 3 and 4. The correlation coefficient between EC and EB is 0.651 at a significance level of 0.01, H1 is supported, i.e., EC and EB are correlated positively and significantly. The correlation coefficient between EC and EI is 0.585 at a significance level of 0.01, so H2 is supported, i.e., EC and EI are correlated positively and significantly. The correlation coefficient between EI and EB is 0.737 at a significance level of 0.01, so H3 is supported, i.e., EI and EB are correlated positively and significantly.

**TABLE 3:** Descriptive statistics.

	Mean	Standard deviation	Number of cases
Gender	1.64	0.480	786
Grade	2.16	0.997	786
School area	4.48	1.983	786
Major category	6.93	3.015	786
Entrepreneurial cognition	2.8271	0.63416	786
Entrepreneurial intention	2.8855	0.94927	786
Recognition perception of entrepreneurship education in colleges and universities	3.0173	0.69238	786
Entrepreneurial behavior	2.5585	0.78881	786

**TABLE 4:** Correlation coefficients.

		Gender	Grade	School area	Major category	Entrepreneurial cognition	Entrepreneurial intention	Recognition perception of entrepreneurship education in colleges and universities	Entrepreneurial behavior
Gender	Pearson correlation	1	−0.117**	0.144**	−0.116**	−0.182**	−0.248**	−0.173**	−0.203**
	Significance (two tailed)		0.001	0.000	0.001	0.000	0.000	0.000	0.000
	Number of cases	786	786	786	786	786	786	786	786
Grade	Pearson correlation	−0.117**	1	−0.170**	−0.048	−0.015	−0.027	0.020	0.019
	Significance (two tailed)	0.001		0.000	0.178	0.680	0.442	0.577	0.597
	Number of cases	786	786	786	786	786	786	786	786
School area	Pearson correlation	0.144**	−0.170**	1	0.022	−0.047	−0.063	−0.133**	−0.064
	Significance (two tailed)	0.000	0.000		0.534	0.185	0.075	0.000	0.073
	Number of cases	786	786	786	786	786	786	786	786
Major category	Pearson correlation	−0.116**	−0.048	0.022	1	0.041	0.083*	0.012	0.037
	Significance (two tailed)	0.001	0.178	0.534		0.255	0.019	0.747	0.298
	Number of cases	786	786	786	786	786	786	786	786
Entrepreneurial cognition	Pearson correlation	−0.182**	−0.015	−0.047	0.041	1	0.585**	0.566**	0.651**
	Significance (two tailed)	0.000	0.680	0.185	0.255		0.000	0.000	0.000
	Number of cases	786	786	786	786	786	786	786	786
Entrepreneurial intention	Pearson correlation	−0.248**	−0.027	−0.063	0.083*	0.585**	1	0.533**	0.737**
	Significance (two tailed)	0.000	0.442	0.075	0.019	0.000		0.000	0.000
	Number of cases	786	786	786	786	786	786	786	786
Recognition perception of entrepreneurship education in colleges and universities	Pearson correlation	−0.173**	0.020	−0.133**	0.012	0.566**	0.533**	1	0.617**
	Significance (two tailed)	0.000	0.577	0.000	0.747	0.000		0.000	0.000
	Number of cases	786	786	786	786	786	786	786	786
Entrepreneurial behavior	Pearson correlation	−0.203**	0.019	−0.064	0.037	0.651**	0.737**	0.617**	1
	Significance (two tailed)	0.000	0.597	0.073	0.298	0.000	0.000	0.000	
	Number of cases	786	786	786	786	786	786	786	786

**Correlation is significant at 0.01 level (two−tailed).

*Correlation is significant at 0.05 level (two−tailed).

### Hypotheses testing

Hierarchical linear regression model was used to test the direct effect, mediating effect and moderating effect proposed by the hypotheses, and the test results are given in Table 5.

**TABLE 5:** Hypotheses testing results.

Variable category		EB M1	EB M2	EB M3	EB M4	EI M5	EI M6
Control variable	Gender	−0.083***	−0.005	−0.283***	−0.057**	−0.083***	−0.005
	Grade	0.015	0.036	0.015	0.016	0.015	0.036
	School area	−0.019	−0.006	−0.019	0.011	−0.019	−0.006
	Major category	0.003	−0.020	0.003	0.009	0.003	−0.020
Independent variable	Entrepreneurial cognition	0.635***	0.333***	0.635***	0.263***	0.635***	0.333***
Mediating variable	Entrepreneurial intention		0.543***	0.543***			
Moderator variable	Recognition perception of entrepreneurship education				0.189**		
Interactive phase	Entrepreneurial cognition*Recognition perception of entrepreneurship education				0.312**	0.067	
Parameters	Observations	786	786	786	786	786	786
	*R*-squared	0.432	0.618	0.432	0.522	0.366	0.421
	Adj_*r*^2^	0.428	0.616	0.428	0.517	0.362	0.416
	Value *F*	118.455	381.592	118.455	73.337	90.089	37.089

*t*-statistic in parentheses. ***p < 0.01, **p < 0.05, *p < 0.1.

First, the direct effect of EC on EB was tested. The control variables were put into the regression model, followed by EC, and the analysis results show that EC has a significant positive impact on EB (M1, β = 0.635, *p* < 0.01), and so H1 is supported.

Second, the mediating effect of EI was examined. The regression results in Table 5 show that EC and EI are correlated positively and significantly (M5, β = 0.635, *p* < 0.01), as are EI and EB (M2, β = 0.543, *p* < 0.001), so H2 and H3 are supported. Then EC and EI were put into the regression model at the same time, and EI and EB were still correlated positively and significantly (M3, β = 0.543, *p* < 0.001), and the effect of EB was reduced from 0.635 to 0.333 (M2, β = 0.333, *p* < 0.01). It can be seen that EI plays a partial intermediary role between EC and EB, so H4 is supported.

Finally, the mediating effect of recognition of college EE on the relationship among EC, EI, and EB was tested. Before the regression analysis of mediating effect, the independent variables and mediating variables were centralized. Model 1 in Table 5 was adopted, which places the control variables in the first layer, places EC in the second layer, and takes EB as the result variable. The results of model 1 show that EB can be affected by EC positively and significantly, and so H1 is further supported. Then, to put model 5 into the first layer as the control variable, to put EC in the second layer, and to put EC, recognition perception of EE in colleges and universities, and mediating effect items to the third layer, the results show that the interaction between EC and EE recognition perception is not significant (M5, β = 0.067, *p* < 0.05), which indicating that the recognition of college EE does not play a regulating role in the relationship between EC and EI; therefore, H5a is not supported. This result is inconsistent with our theoretical expectation, i.e., the recognition perception of EE may not necessarily strengthen the positive relationship between EC and EI. The empirical study based on the theory of planned behavior shows that EE can indeed improve EC and enhance EI by teaching basic entrepreneurial knowledge and carrying out practical education. However, students’ EI is determined by not only EE. Regardless of a person’s recognition and perception of EE, their inherent attitude, subjective norms, beliefs, and behavior control before receiving EE can affect their EI most significantly. Also, empirical research based on the entrepreneurial event model shows that because of the relatively weak predictive ability of the antecedent variables related to EI—such as subjective norms, desirability, and feasibility perception—and the difficulty of making accurate measurements in practice, even though EE courses have a positive effect on a person’s perception of feasibility, the effect on their perception of desirability is not significant, i.e., EE may have a significant effect on individual entrepreneurial self-efficacy, but its relationship with EI is still weak (Xiu’e and Kun, 2016). Therefore, in a complex reality, individuals’ recognition perception of EE does not always reach an appropriate level and will be affected by the perception of feasibility and desirability. The specific differences of EE on EC and EI also need to be explored further.

According to the above methods, the mediating effect of the recognition of college EE on the relationship between EC and EB was tested. Model 1 in Table 5 was adopted, which places the control variables in the first layer, places EC in the second layer, and takes EB as the result variable. The results of model 1 show that EB can be positively predicted by EC, and so H1 is further supported. Finally, model 2 puts the control variables in the first layer, EC in the second layer, and EC, recognition of college EE, and mediating effect items in the third layer. The results show that the interaction between them also has a significant impact on EB (M4, β = 0.312, *p* < 0.05). Therefore, it can be concluded that the recognition of university EE plays a positive mediating role in the relationship between EC and EB. Therefore, H5b is supported, i.e., the higher the degree of college students’ perception of the recognition of EE in colleges and universities, the stronger the positive relationship between their EC and EB.

## Conclusions and recommendations

### Conclusion

This study explored the impact mechanism of college students’ EC on their EB, and the results showed the following. (1) EC can significantly and positively influence the EB of college students. (2) EC can significantly and positively influence the EI of college students. (3) There is a significant positive correlation between the EI of college students and their EB. (4) The EI of college students partially mediates the positive correlation between their EC and EB, i.e., college students’ EC affects their EB by affecting their EI. (5) The relationship between the recognition of college EE and EC and EI did not exert a significant positive moderating effect, but it played a significant positive moderating effect on the relationship between EI and EB, i.e., high-quality EE can stimulate further EB of college students. Therefore, the focus of college EE reform should be on how to reform and innovate entrepreneurship cognitive education to stimulate its positive impact on the EI and EB of the existing college students.

### Theoretical contributions

First, the present study breaks through the limitations of previous EE research focusing on the common EB of college students and single cognitive perspective, which pays more attention to the impact of entrepreneurial cognitive education on the change of college students’ individual personality and traits on their perceived behavior control. Based on the behavioral logic of individual “cognition–emotion–intention–behavior,” this study focuses on the relationship between EC and EB and responds to the previous research conclusions of scholars such as Fayolle, Peterman, Kennedy, and Zhang: EE may have little effect on the entrepreneurial enthusiasm of college students, and in individual cases it may have no effect or even a negative impact on EI and its antecedent variables.

Second, in combination with the new trend of mass entrepreneurship and innovation in China in recent years, this manuscript conducted research from the perspective of the specific content and mode of EE reform in colleges and universities, integrated the recognition of college EE into the research framework of college students’ EC and EB, and supplemented and integrated the previous theories based on planned-behavior theory, dual cognitive processing theory, self-efficacy theory, or the entrepreneurial event model, i.e., by revealing the boundary conditions of the role of EC, it expands the research perspective of college students’ EB. Previous theoretical and empirical studies have shown that if a college or university provides EC and practical support, then the possibility of college students choosing entrepreneurship will increase greatly. However, these studies ignore the audience of EE: college students’ own individual characteristics, their perception of entrepreneurial feasibility, their perception of acceptability, and their perception of recognition of the existing EE. Our research results show that in the current era of mass entrepreneurship and innovation, college students’ EB is affected comprehensively by self-EC, individual EI, and recognition perception of EE in colleges and universities. It is shown that the recognition perception of EE in colleges and universities is an important boundary condition for college students’ EC to have a positive effect on their EB.

Third, as a pre-factor of individual behavior, EI mediates the relationship between college students’ EC and their EB. On one hand, EC can significantly promote college students’ EI and stimulate self-efficacy, and the strengthening of EI further promotes college students’ EB. On the other hand, note that the EI of college students is not entirely determined by the EE in colleges and universities. The individual’s inherent attitude toward entrepreneurship, subjective norms, previous experience, and perceived behavior will also affect their EI, i.e., the recognition of college EE does not necessarily have a completely significant positive effect on the EI of individual college students, and the EI of individual college and university students who have received EE in colleges and universities may also be reduced.

### Research implications

In EE in colleges and universities, there is a natural connection among EC, EI, and EB, and the continuous accumulation of entrepreneurial knowledge and experience can gradually be transformed into entrepreneurial practice ability. However, EI is endogenous and exogenous, including the impact of cognition of independence, challenges, achievements, rights, wealth, interests, habits, efficacy, family, education, and social recognition on the individual differentiation. At the same time, the factors that affect EC are also complex. EE in colleges and universities is only one factor that affects EC, and there may be other factors, so the recognition perception of EE in colleges and universities may not strengthen the positive relationship between EC and EI. However, the highly recognized EE perception in colleges and universities can significantly promote college students’ EB. Therefore, EE in colleges and universities can directly affect the EB of college students by constructing a scientific and operable EE model. Therefore, the focus of subsequent research will be on how to reform and innovate the existing college EE model to stimulate the positive impact of college students’ EC on their EI and EB. Furthermore, the main body of entrepreneurship education is not only universities and colleges, families, entrepreneurial enterprises, government, entrepreneur and other entrepreneurial stakeholders should be involved in, and then to play, respectively, effect to help students processing EC positively.

### Research limitations

In the present research, college and university students’ individual self-assessment was used to measure their EC and recognition of college EE. However, although person always thinks that he or she knows himself or herself best, there may still have been social-approval bias and common-method bias, which may have affected the objectivity of the research results. In future work, a combination of self-assessment and other assessment could be chosen for measurement.

The research on EC itself involves many variables and their dimension choices. This study only verified its impact on EI and EB from the two dimensions of entrepreneurial-readiness cognition and entrepreneurial-ability cognition, which is not comprehensive enough. As a pre-variable of entrepreneurial intention, the positive effect of strengthening entrepreneurial cognition on EI and EB of entrepreneurship education in colleges and universities may need to be further explored. Also, there is still a lack of cultivation and tracking research on the EC of potential entrepreneurs.

## Data availability statement

The raw data supporting the conclusions of this article will be made available by the authors, without undue reservation.

## Author contributions

XT conceptualized the contribution. SC and WS wrote and reviewed the manuscript. RL made critical revisions. All authors approved the submission of the manuscript.
